# Recurrence of gastric cancer with Krukenberg tumor, presenting with a high alpha-fetoprotein level, a case report

**DOI:** 10.3389/fonc.2026.1676891

**Published:** 2026-02-19

**Authors:** Elif Haznedaroglu Benlioglu, Samed Rahatli, Seyfettin Baltacioglu, Mehmet Burkay Karakulak, Efe Yetisgin, Mustafa Altinbas

**Affiliations:** Ankara Etlik City Hospital, Ankara, Türkiye

**Keywords:** alpha fetoprotein (AFP), gastric cancer, Krukenberg metastases, metastatic gastric cancer, ovarian metastases

## Abstract

Gastric cancer is a common malignancy globally, often presenting with advanced disease and a propensity for metastasis. While peritoneal dissemination and liver involvement are frequent metastatic sites, ovarian metastasis, particularly Krukenberg tumors, represents a less common but significant manifestation, typically associated with a poor prognosis. The presence of elevated tumor markers, such as carcinoembryonic antigen (CEA) and CA19-9, is often indicative of advanced disease. However, the elevation of Alpha-fetoprotein (AFP) in gastric cancer, a marker more commonly associated with hepatocellular carcinoma or germ cell tumors, is exceedingly rare and suggests an unusual biological behavior or specific histological subtypes. This case report details the presentation of a 30-year-old female patient with gastric adenocarcinoma that metastasized to the ovaries, characterized by a significant elevation in serum AFP levels. This case highlights the critical need to consider uncommon metastatic patterns and unusual tumor marker elevations during the comprehensive evaluation and management of gastric cancer patients.

## Introduction

Gastric cancer is a globally common malignancy which has a high mortality rate (1). Despite advances in diagnosis and treatment, diagnoses at advanced stages are common, which leads to a poor prognosis (2).

Alpha-fetoprotein is a glycoprotein which is normally found in the fetal liver and yolk sac (3). Elevation of alpha-fetoprotein level is considered abnormal in adults, and it is a distinct tumor marker for yolk sac tumor and hepatocellular carcinoma (4). Alpha-fetoprotein level was also shown to be produced in colorectal cancer and lung cancer (5–8). Various case reports, case series and studies show elevated serum alpha-fetoprotein as a significant biomarker in gastric cancer, correlating especially with liver metastases, venous invasion, lymphatic invasion and an overall poorer prognosis (9–12). Krukenberg tumor, the metastatic spread of gastric adenocarcinoma to the ovary, is an uncommon yet clinically critical presentation of late-stage disease (13).

This case report details the presentation, diagnosis, and management of a 30-year-old female patient with gastric adenocarcinoma that metastasized to the ovaries, uniquely characterized by a significant elevation in serum AFP levels. This case underscores the importance of considering rare metastatic patterns and atypical tumor marker elevations in the comprehensive evaluation and management of gastric cancer patients.

## Case presentation

A 30-year-old female patient with a history of gastric adenocarcinoma presented with vomiting, anorexia, dysphagia and was admitted to the oncology ward. Her complaints started two weeks ago and gradually increased. She had no other chronic diseases and had a 12-pack-year smoking history. The patient underwent subtotal gastrectomy a year prior. Because of intolerance she could only receive one cycle of neoadjuvant, and four cycles of adjuvant Docetaxel-cisplatin-5-fluorouracil (DCF) chemotherapy. (Despite clinical recommendations, the patient had declined to continue treatment due to severe treatment-induced nausea and vomiting in the neoadjuvant setting.) Family history was unremarkable for gastric or ovarian malignancies. Genetic testing for CDH1 mutations could not be performed due to institutional limitations.

She endorsed that she felt lower abdominal pain and bloating. Her ECOG (Eastern Cooperative Oncology Group) performance status was 1 upon admission. Physical examination revealed a laparotomy scar and an approximately 10-cm mass in the lower abdominal area. No ascites was present in the physical examination. Laboratory results revealed anemia (10 g/dL) and hypoalbuminemia (2.7 g/L). Tumor markers were notable for an elevated Alpha-fetoprotein (AFP) level of 7850 ng/mL, while CEA, CA19–9 and CA72–4 levels were within normal limits.

**Figure 1:**
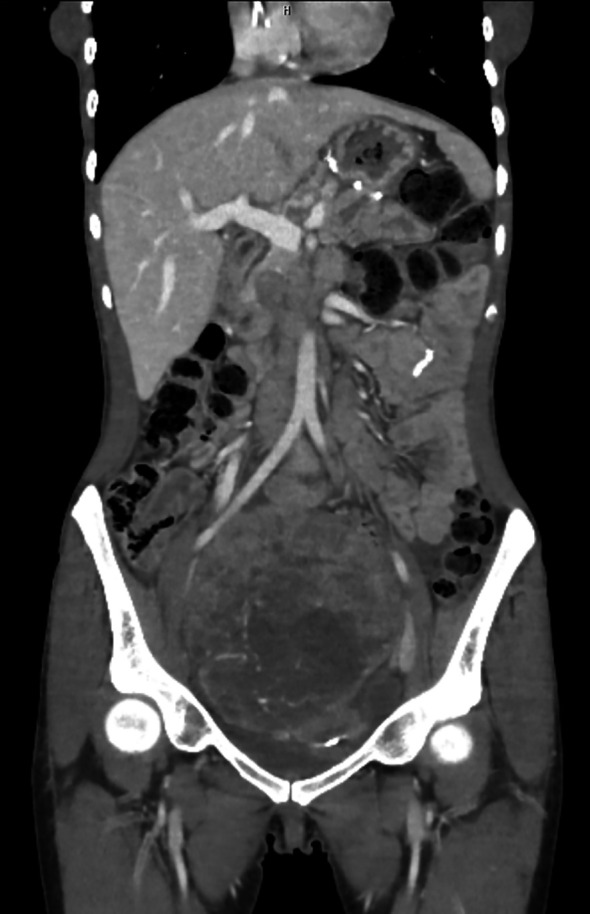
Computed tomography scan of the patient. An abdominal computed tomography (CT) scan showed a 135x120 mm, well-circumscribed, heterogeneous mass filling the pelvic region. It displayed intense heterogeneous contrast enhancement with central hypodense areas suggestive of necrosis and compressed both bilateral external and internal iliac veins. No liver metastasis or peritoneal implants were observed.

An abdominal computed tomography (CT) scan showed a 135x120 mm, well-circumscribed, heterogeneous mass filling the pelvic region. It displayed intense heterogeneous contrast enhancement with central hypodense areas suggestive of necrosis and compressed both bilateral external and internal iliac veins. Multiple lymph nodes (LAP) are noted in the right internal iliac region, largest of which is 22x13 mm in size. No liver metastasis, other lymph node involvements, bladder-rectal invasion or peritoneal implants were observed (**Figure 1**).

Given the elevated AFP, and after consultation with gynecologic and surgical oncology, a surgical exploration was planned to palliate symptoms and investigate preliminary diagnoses of a gynecological secondary malignancy or Krukenberg tumor.

**Figure 2:**
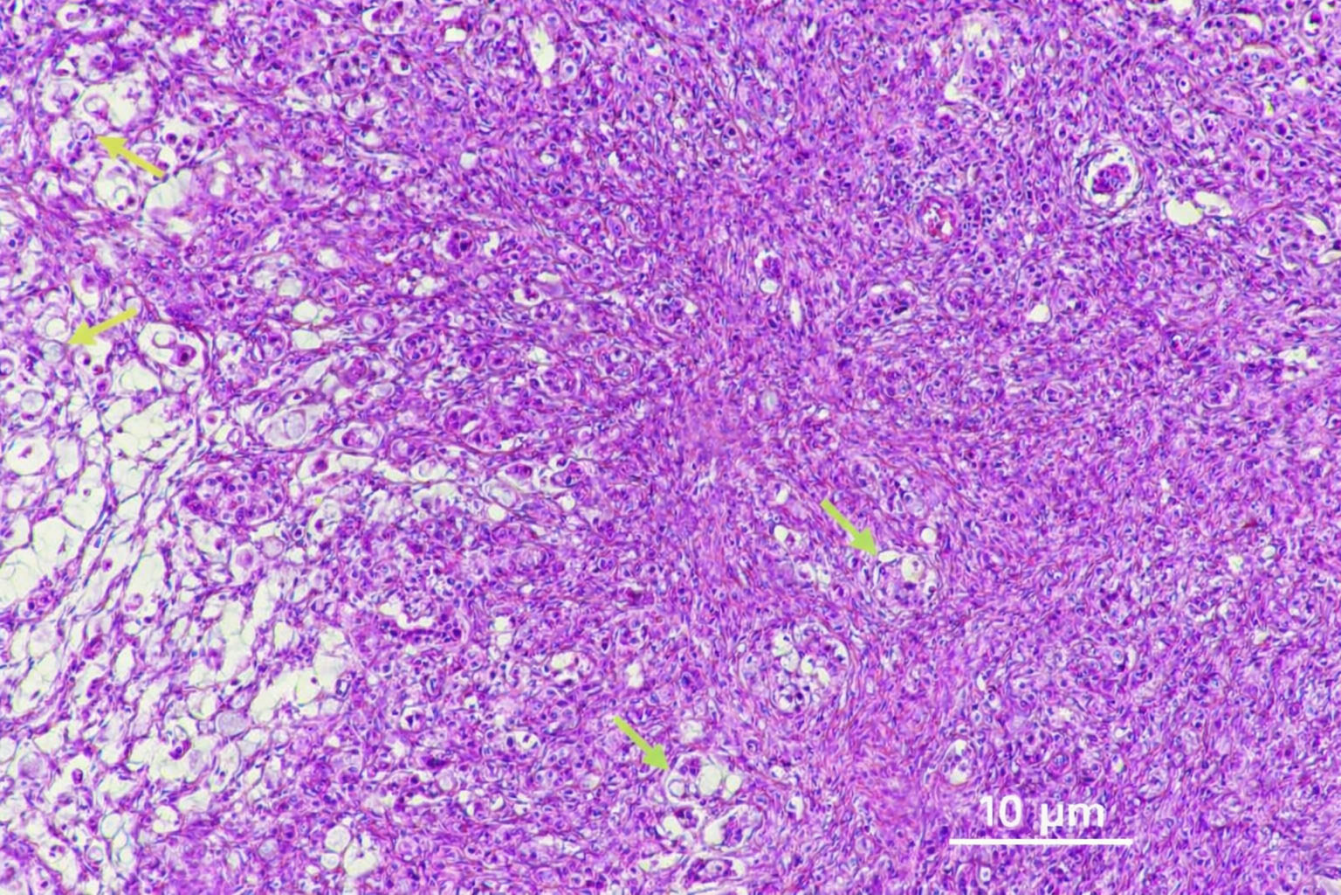
Microscopic histopathology of the surgical specimen. High-power photomicrograph (hematoxylin and eosin, ×100) showing malignant foveolar cells with eosinophilic cytoplasm, atypical nuclei, and prominent nucleoli. Green arrows: malignant foveolar glands; yellow arrows: signet-ring cell morphology.

During the exploratory laparotomy, metastatic implants were found in multiple locations, including the small bowel loops, mesentery, and the Y-anastomosis limb. A 10 cm mass originating from the right ovary was identified in the right lower quadrant, pulling both ovarian structures towards itself, consistent with a Krukenberg tumor. A biopsy was taken, and the patient was monitored post-operatively in the intensive care unit. The biopsy confirmed malignant cells with eosinophilic cytoplasm and atypical nuclei and nucleoli within the ovarian stroma, consistent with metastasis of gastric cancer (**Figure 2**). Following surgical exploration, the patient unfortunately succumbed to postoperative infectious complications.

## Timeline

In September 2023, the patient was diagnosed with gastric adenocarcinoma following endoscopic evaluation. After the initial cycle of Docetaxel, Cisplatin, and 5-Fluorouracil (DCF) chemotherapy, the treatment was discontinued due to intractable chemotherapy-induced nausea and vomiting. The patient subsequently underwent a subtotal gastrectomy in October 2023, followed by the administration of four adjuvant DCF cycles.

In early December 2024, the patient presented with progressive symptoms of emesis, anorexia, and dysphagia, leading to admission to the medical oncology department on December 12, 2024. Although endoscopic assessment revealed no evidence of recurrence or external compression at the anastomotic site, computed tomography (CT) imaging identified a massive pelvic mass. Laboratory investigations were unremarkable, with the exception of mild anemia, hypoalbuminemia and an elevated alpha-fetoprotein (AFP) level. Following surgical exploration in late December 2024, the patient unfortunately succumbed to postoperative infectious complications.

## Discussion

Ovarian metastasis from gastric cancer, commonly known as a Krukenberg tumor, is a relatively rare but clinically significant manifestation of advanced gastric adenocarcinoma. While the gastrointestinal tract, particularly the stomach, colon, and appendix, are the most frequent primary sites, gastric cancer accounts for a substantial proportion of these metastases, typically indicating an advanced stage and a poor prognosis (13). In this case, the presence of a 10 cm right ovarian mass, along with widespread peritoneal implants on small bowel loops, mesentery, and the Y-anastomosis limb, unequivocally confirmed the metastatic nature of the disease, consistent with a Krukenberg tumor. The biopsy findings of malignant cells with eosinophilic cytoplasm and atypical nuclei in the ovarian stroma further supported the gastric origin.

A striking feature of this patient’s presentation was the significantly elevated serum Alpha-fetoprotein (AFP) level of 7850 ng/mL. AFP is primarily recognized as a tumor marker for hepatocellular carcinoma and germ cell tumors (4). Its elevation in gastric cancer is considered rare, reported in approximately 1-10% of cases, and often suggests a distinct biological subtype, such as hepatoid adenocarcinoma or gastric cancer with enteroblastic differentiation (14, 15).

The clinical implications of AFP elevation in gastric cancer are profound. Numerous studies have demonstrated that AFP-producing gastric cancers are often associated with more aggressive biological behavior, including a higher incidence of liver metastasis, deeper tumor invasion, increased lymphatic and venous invasion, and a generally poorer prognosis compared to non-AFP-producing gastric cancers (9, 10). The patient’s severe symptoms (vomiting, anorexia, dysphagia), the large pelvic mass, and the extensive metastatic implants observed during laparotomy are consistent with the aggressive nature often seen in AFP-producing gastric cancers. Furthermore, elevated AFP levels in gastric cancer have been linked to a reduced response to conventional chemotherapy regimens (16). The diagnostic work-up, including the abdominal CT scan and comprehensive tumor marker panel, was crucial in identifying the extensive pelvic mass and the atypical AFP elevation, which guided the decision for surgical exploration. Despite the established association between AFP elevation and liver metastases in gastric cancer (9–12), our patient’s CT scan notably showed no evidence of liver involvement. Instead, the sole finding was a large pelvic mass, which initially led us to consider a secondary germ cell tumor in the differential diagnosis. While the surgery in this advanced setting was primarily palliative, aimed at alleviating the patient’s symptoms, it provided definitive histological confirmation of the Krukenberg tumor and the widespread metastatic burden. This case underscores the importance of a thorough diagnostic evaluation, including a broad tumor marker panel, even in patients with known primary malignancies, as unusual marker elevations can provide critical insights into disease behavior and prognosis.

In conclusion, this case highlights a rare presentation of gastric adenocarcinoma with ovarian metastasis (Krukenberg tumor) accompanied by an exceptionally high serum AFP level. This unusual combination emphasizes the need for clinicians to consider atypical metastatic patterns and uncommon tumor marker elevations in the comprehensive evaluation and management of gastric cancer patients, as these findings often correlate with aggressive disease and a challenging prognosis.

## Conclusions

This case report underscores the critical importance of a comprehensive diagnostic approach in gastric cancer, particularly when faced with atypical presentations. The occurrence of a Krukenberg tumor with an exceptionally high Alpha-fetoprotein (AFP) level, despite the absence of liver metastasis, highlights the unpredictable and aggressive nature of certain gastric adenocarcinoma subtypes. Clinicians should remain vigilant for unusual metastatic patterns and uncommon tumor marker elevations, as these findings can provide invaluable insights into disease behavior, prognosis, and ultimately guide more tailored management strategies for patients with this challenging malignancy.

## Data Availability

The original contributions presented in the study are included in the article/supplementary material. Further inquiries can be directed to the corresponding author.

## References

[1] Ferlay J Colombet M Soerjomataram I Soerjomataram I Mathers C Parkin DM . Estimating the global cancer incidence and mortality in 2018: GLOBOCAN sources and methods. Int J Cancer. (2019) 144:1941–53. doi: 10.1002/ijc.31937, PMID: 30350310

[2] Smyth EC Nilsson M Grabsch HI Van Grieken NC Lordick F . Gastric cancer. Lancet. (2020) 396:635–48. doi: 10.1016/S0140-6736(20)31288-5, PMID: 32861308

[3] Wang Y Zhang S Ding B Tang Z Ji Y Yu Y . Development and validation of an individualized nomogram for gastric cancer patients treated with perioperative chemotherapy followed by radical surgery. Transl Gastroenterol Hepatol. (2024) 9:39. doi: 10.21037/tgh-23-75, PMID: 39091661 PMC11292059

[4] Wepsic HT Kirkpatrick A . Alpha-fetoprotein and its relevance to human disease. Gastroenterology. (1979) 77:787–96. doi: 10.1016/0016-5085(79)90238-5 89060

[5] Feng Y Li Y Dai W Mo S Li Q Cai S . Clinicopathologic features and prognostic factors in alpha-fetoprotein-producing colorectal cancer: Analysis of 78 cases. Cell Physiol Biochem. (2018) 51:2052–64. doi: 10.1159/000495824, PMID: 30522102

[6] Ren F Weng W Zhang Q Tan C Xu M Zhang M . Clinicopathological features and prognosis of AFP-producing colorectal cancer: A single-center analysis of 20 cases. Cancer Manag Res. (2019) 11:4557–67. doi: 10.2147/CMAR.S196919, PMID: 31191017 PMC6529609

[7] Zhuansun Y Bian L Zhao Z Du Y Chen R Lin L . Clinical characteristics of Hepatoid adenocarcinoma of the lung: Four case reports and literature review. Cancer Treat Res Commun. (2021) 29:100474. doi: 10.1016/j.ctarc.2021.100474, PMID: 34656923

[8] Ding H Xu S Wang K Wang X Sun G Li X . Alpha-fetoprotein-producing advanced colorectal cancer: A rare case report and literature review. J Int Med Res. (2022) 50:3000605221117218. doi: 10.1177/03000605221117218, PMID: 35999811 PMC9421241

[9] Zhan Z Chen B Yu J Zheng J Zeng Y Sun M . Elevated serum alpha-fetoprotein is a significant prognostic factor for patients with gastric cancer: results based on a large-scale retrospective study. Front Oncol. (2022) 12:901061. doi: 10.3389/fonc.2022.901061, PMID: 35847953 PMC9277009

[10] Takayama-Isagawa Y Kanetaka K Kobayashi S Yoneda A Ito S Eguchi S . High serum alpha-fetoprotein and positive immunohistochemistry of alpha-fetoprotein are related to poor prognosis of gastric cancer with liver metastasis. Sci Rep. (2024) 14:3695. doi: 10.1038/s41598-024-54394-1, PMID: 38355790 PMC10866906

[11] Zuo F Tan Y Mao W Tang Z Luo T . Clinical prognosis evaluation of alpha-fetoprotein-positive gastric cancer: comprehensive analysis and development of a novel nomogram for survival prediction. Front Oncol. (2025) 15:1598337. doi: 10.3389/fonc.2025.1598337, PMID: 40485723 PMC12141012

[12] Chun H Kwon SJ . Clinicopathological characteristics of alpha-fetoprotein-producing gastric cancer. J Gastric Cancer. (2011) 11:23–30. doi: 10.5230/jgc.2011.11.1.23, PMID: 22076198 PMC3204474

[13] Kiyokawa T Young RH Scully RE . Krukenberg tumors of the ovary: A clinicopathologic analysis of 120 cases with emphasis on their variable pathologic manifestations. Am J Surg Pathol. (2006) 30:277–99. doi: 10.1097/01.pas.0000190787.85024.cb, PMID: 16538048

[14] Abe D Akazawa Y Yatagai N Hayashi T Ueyama H Mine S . Clinicopathological characteristics of gastric adenocarcinoma with enteroblastic differentiation and gastric adenocarcinoma with enteroblastic marker expression. Virchows Arch. (2023) 483:405–14. doi: 10.1007/s00428-023-03623-5, PMID: 37581693

[15] Liu X Cheng Y Sheng W Lu H Xu X Xu Y . Analysis of clinicopathologic features and prognostic factors in hepatoid adenocarcinoma of the stomach. Am J Surg Pathol. (2010) 34:1465–71. doi: 10.1097/PAS.0b013e3181f0a873, PMID: 20871221

[16] Wang YK Shen L Jiao X Zhang XT . Predictive and prognostic value of serum AFP level and its dynamic changes in advanced gastric cancer patients with elevated serum AFP. World J Gastroenterol. (2018) 24:266–73. doi: 10.3748/wjg.v24.i2.266, PMID: 29375212 PMC5768945

